# Tuning the Properties of 3D‐Printed Electrochemical Devices by Using Different Fused Deposition Modeling Printers and Setups

**DOI:** 10.1002/open.70303

**Published:** 2026-09-24

**Authors:** Gennaro Persico, Panagiota M. Kalligosfyri, Elena Lagreca, Vincenzo Ianniello, Concetta Di Natale, Michela Volgare, Antonio Cutolo, Filippo Defina, Waleed Alahmad, Abdulaziz K. Assaifan, Mariarosaria Bucci, Stefano Cinti

**Affiliations:** ^1^ Department of Pharmacy University of Naples Federico II Naples Italy; ^2^ Dipartimento di Ingegneria Chimica, dei Materiali e della Produzione Industriale University of Naples Federico II Naples Italy; ^3^ 3DnA s.r.l. Naples Italy; ^4^ Department of Chemistry Faculty of Science Chulalongkorn University Bangkok Thailand; ^5^ Biomedical Technology Department College of Applied Medical Sciences King Saud University Riyadh Saudi Arabia; ^6^ Sbarro Institute for Cancer Research and Molecular Medicine Center for Biotechnology College of Science and Technology Temple University Philadelphia Pennsylvania USA; ^7^ Bioelectronics Task Force at University of Naples Federico II Naples Italy

**Keywords:** 3D‐printed device, 3D‐printing, additive manufacturing, characterization, electrochemical sensors

## Abstract

Although fused deposition modeling (FDM) 3D printing has emerged as an accessible and versatile approach for the fabrication of electrochemical sensors, the type of printer and manufacturing setup might affect the quality of the ultimate device. In this work, the electrochemical performance of conductive polylactic acid (PLA)‐based printed sensors have been tuned by interrogating different cost classes and hardware configurations. Electrochemical behavior was investigated using cyclic voltammetry with both inner‐ and outer‐sphere redox probes, namely potassium ferricyanide (III) and hexaammineruthenium (III) chloride, to evaluate surface accessibility and electron‐transfer properties. Scanning electron microscopy and tensile tests were additionally performed to characterize electrode morphology and mechanical stability. Among the evaluated 3D printers, high‐performance systems characterized by higher automation, improved motion control, and greater extrusion stability demonstrated satisfactory outcomes. In contrast, entry‐level open‐platform systems showed lower surface utilization and reduced electrochemical performance. The results demonstrate that all printing platforms were capable of producing sensors with electrochemical functionality, comparable mechanical properties, and well‐defined architectures. However, despite negligible differences among PLA filaments, it is clear that printing time, automation, and hardware flexibility represent central factors in selecting the most suitable platform for next scaling of electrode manufacturing.

## Graphical abstract

Four fused‐deposition modeling printers spanning entry‐level to industrial platforms were compared for conductive PLA electrode fabrication. Electrochemical, morphological, mechanical, time, and cost analyses show that printer automation and hardware configuration influence electrode performance, while all platforms can produce high‐quality sensors.

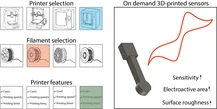

## 1 Introduction

Additive manufacturing has emerged as one of the most transformative enabling technologies in analytical chemistry, offering unprecedented flexibility in the design and fabrication of electrochemical and biosensors. Among the various additive manufacturing strategies, techniques such as stereolithography, digital light processing, inkjet printing, and fused deposition modeling (FDM) have been explored for the fabrication of electrochemical devices [1, 2]. Specifically, FDM has emerged as the most accessible and widely adopted fabrication route, combining low instrumentation costs with the ability to produce complex, customizable geometries without the need for specialized infrastructure [3]. In FDM technology, a thermoplastic filament is melted and extruded through a heated nozzle, depositing the material layer by layer according to a digital design file. This process directly enables rapid prototyping cycles and decentralized production capabilities, which are particularly well‐suited for research and point‐of‐care applications [2, 4].

A key driver behind the rapid growth of FDM‐based electrochemical sensing has been the parallel development of conductive thermoplastic filaments. Commercially available and laboratory‐prepared composites, including polylactic acid (PLA) loaded with carbon black, graphene, or carbon nanotube additives, have dramatically expanded the palette of printable electrode materials [5, 6, 7]. These filaments offer tunable conductivity and electrochemical activity, and their increasing availability has stimulated a wave of innovation in electroanalysis and biosensing, spanning clinical diagnostics [3, 8, 9, 10], environmental monitoring [11], and agri‐food control [12, 13]. The design flexibility offered by FDM printing can be further exploited through chemometric and data‐driven optimization approaches to rapidly identify optimal electrode geometries and printing parameters [14]. Moreover, the compatibility of 3D‐printed electrodes with emerging substrates, including paper‐based microfluidics and soft, flexible materials, opens new opportunities for integrated, wearable, and decentralized electrochemical sensing systems [15].

Despite these advantages, the electrochemical performance of FDM‐printed sensors is not determined solely by filament composition. Because FDM is inherently a layer‐by‐layer deposition process, the final electrode architecture, and therefore its analytical behavior, is critically shaped by a set of interdependent printing parameters: layer thickness and deposition geometry, interlayer adhesion, the continuity of conductive pathways, surface roughness, porosity, and the density of exposed electroactive domains at the electrode–solution interface [16, 17, 18]. Modulating these parameters is critical for optimizing electrode performance, as it enables precise control over the active surface area, mass transport characteristics, conductive network accessibility, and electron‐transfer behavior [17, 19, 20, 21]. However, a fundamental challenge remains largely unaddressed in the field: the lack of manufacturing standardization across different laboratories, particularly due to the use of diverse 3D printer models, technologies, and cost ranges, which may introduce significant variations in fabrication outcomes and sensor or component performance [22, 23]. Different research groups operate with different FDM printers, including low‐cost desktop systems such as Creality Ender 3 [8, 24], advanced consumer/prosumer platforms such as Bambu Lab P1S and Prusa i3 MK3S+, as well as professional‐grade systems such as Ultimaker S5 and Raise3D Pro3. These platforms differ in mechanical precision, extrusion systems, automation level, calibration procedures, and process stability, which can introduce variability in fabrication outcomes and ultimately affect electrode performance and reproducibility. Printer hardware differs substantially in ways that are rarely reported or controlled, such as motion systems (Cartesian, CoreXY, and delta), positioning accuracy, vibration damping, temperature regulation of both nozzle and build plate, and the specific slicing software and parameter implementations used to translate digital models into physical layers [25]. Consequently, nominally identical electrodes fabricated from the same conductive filament but using different FDM printing platforms may exhibit differences in their electrochemical properties, potentially affecting reproducibility and complicating comparisons across laboratories. The present work aims to systematically investigate this underexplored source of variability.

Here, we report a systematic comparison of FDM‐printed electrochemical sensors produced using four different classes of 3D printers, categorized according to acquisition cost, hardware configuration, and degree of automation: Bambu Lab X1‐Carbon (X1‐Carbon), Bambu Lab A1 Mini (A1 Mini), Creality Ender‐3 V2 Neo (Creality), and FlashForge Creator 4‐HS (HS). We evaluate how differences in printer hardware and printing processes influence key electrochemical metrics, including electroactive surface area (ECSA), peak‐to‐peak separation, peak current, roughness factor, and electrode‐to‐electrode reproducibility. Mechanical integrity and surface morphology were additionally assessed through tensile testing and scanning electron microscopy (SEM). Our results demonstrate that while all platforms yielded viable electrodes with comparable mechanical properties and cost‐effective fabrication (~0.013 €/electrode), electrochemical performance was printer‐dependent and influenced by the redox probe employed, with Creality consistently underperforming in terms of surface accessibility across both inner‐ and outer‐sphere redox probes. These findings provide researchers with an evidence‐based framework for selecting appropriate FDM printing platforms for electrochemical sensor fabrication, enabling informed decisions based on laboratory budget, required printing resolution, desired sensor performance, and practical operational constraints.

## 2 Experimental Section

### 2.1 Reagents and Equipment

Detailed information on the materials, equipment specifications, and 3D‐printing parameters used in this study is provided in the Supporting Information.

### 2.2 Fabrication of the 3D‐Printed Electrochemical Electrodes

The 3D‐printed electrodes were designed in the Autodesk Fusion design application with the following dimensions: 4 mm width, 2 mm thickness, and 13.5 mm height, as reported in Figure S1A. The design was then converted to 3MF or STL format and imported into the corresponding slicer software: UltiMaker Cura 5.4.0 for the Creality, FlashPrint 5.8.7 for the HS, and Bambu Studio 2.4.0.70 for the Bambu Lab printers. A line infill pattern with 100% infill density, a nozzle temperature of 230 °C, and a printing bed temperature of 55 °C were maintained across all platforms. Printer‐specific motion and speed parameters, including printing speed, acceleration, first‐layer speed, infill speed, external wall speed, and top‐layer speed, were retained according to the corresponding printer/slicer configuration and are reported in Table S1. Before use, the 3D‐printed electrodes were pretreated with a 0.5 M solution of NaOH [26, 27]. A potential of 1.4 V was applied for 200 s, followed by a −1.0 V potential for another 200 s. Finally, the electrodes were rinsed with distilled water. The reference and counter electrodes were externally fabricated screen‐printed electrodes (SPEs) manually printed on a flexible polyester substrate using conductive inks, as described previously [28]. Briefly, Ag/AgCl ink was used for the reference electrode and graphite‐based ink for the counter electrode. The SPEs were sequentially treated in an oven at 100 °C for 30 min after each printing step. Commercial adhesive tape was used to insulate printed electrodes. For the electrochemical measurements, *n* = 3 corresponds to three independently fabricated electrodes for each printing platform, with each electrode produced in a separate printing run/batch using the corresponding 3D printer under the same predefined printing conditions.

### 2.3 Evaluation of Electrodes’ Mechanical Properties via Tensile Analysis

Details regarding specimen preparation, testing conditions, and data analysis procedures are provided in the Supporting Information.

### 2.4 SEM

Further details regarding the SEM analysis are provided in the Supporting Information.

### 2.5 EDX Elemental Analysis

The elemental composition of the 3D‐printed carbon electrodes was characterized by energy‐dispersive X‐ray spectroscopy (EDX) coupled with SEM. Further details regarding the EDX analysis, including representative spectra and elemental maps, are presented in the Supporting Information.

### 2.6 Electrochemical Characterization

Electrochemical characterization was performed using hexaammineruthenium (III) chloride ([Ru(NH_3_)_6_]Cl_3_) and potassium ferricyanide (III) (K_3_[Fe(CN)_6_]) in cyclic voltammetry (CV), respectively, inner‐sphere probe (i.e., more strongly influenced by surface chemistry) and outer‐sphere probe (i.e., less influenced by surface chemistry). Potassium ferricyanide was prepared at a concentration of 1 mM in 1 M KCl. The CV was performed with scan rates ranging from 10 to 200 mV s^−1^, E_vertex between −0.6 and 1.0 V, and E_step 0.01 V. Hexammineruthenium (III) chloride was prepared at a concentration of 1 mM in 1 M KCl. The CV was performed with scan rates ranging from 10 to 200 mV s^−1^, E_vertex between −0.6 and +0.5 V, and E_step 0.01 V. For all analyses, the electrodes were immersed in a vertical configuration in a 3D‐printed microwell with a capacity of 700 µL (Figure S1). To evaluate the electrochemical performance of the 3D‐printed electrodes fabricated by the four different printers (X1‐Carbon, A1 Mini, Creality, and HS), the electrochemically active surface area was estimated using the Randles–Ševčík equation under diffusion‐controlled conditions. The peak current was plotted against the square root of the scan rate, and the slope of the resulting linear relationship was used to calculate the ECSA. For potassium ferricyanide, a diffusion coefficient of 7.6 × 10^−^^6^ cm^2^ s^−1^ was used, as reported in the literature [29], while a diffusion coefficient of 8.8 × 10^−^^6^ cm^2^ s^−1^ was used for hexaammineruthenium(III), according to the literature [30]. In both cases, a one‐electron transfer (*n* = 1) and a redox probe concentration of 1 mM were considered. The ECSA was calculated from the slope of the peak current versus the square root of the scan rate using the standard Randles–Ševčík relationship. The corresponding plots are provided in Figures S6 and S7 for potassium ferricyanide and hexaammineruthenium(III), respectively. The obtained linear relationships showed high coefficients of determination (*R* ^2^ = 0.97–0.99), supporting the diffusion‐controlled approximation used for the comparative ECSA estimation. Given the quasireversible behavior of the printed electrodes, the calculated electroactive area values are considered comparative estimates of the electrochemically accessible area rather than absolute physical surface‐area measurements. In the present work, the roughness factor was calculated as the ratio of the electrochemically active surface area and the geometric area of the electrode. Therefore, the roughness factor represents the fraction of the nominal geometric electrode area that is electrochemically accessible to the redox probe under the experimental conditions. In this context, it should be interpreted as an electrochemical accessibility parameter rather than as a direct measurement of the physical surface roughness. The total geometric surface area (Ageom) for the exposed 3D cylindrical working electrode (radius = 2 mm and height = 2 mm) was established at 0.5027 cm^2^.

## 3 Results and Discussion

**FIGURE 1:**
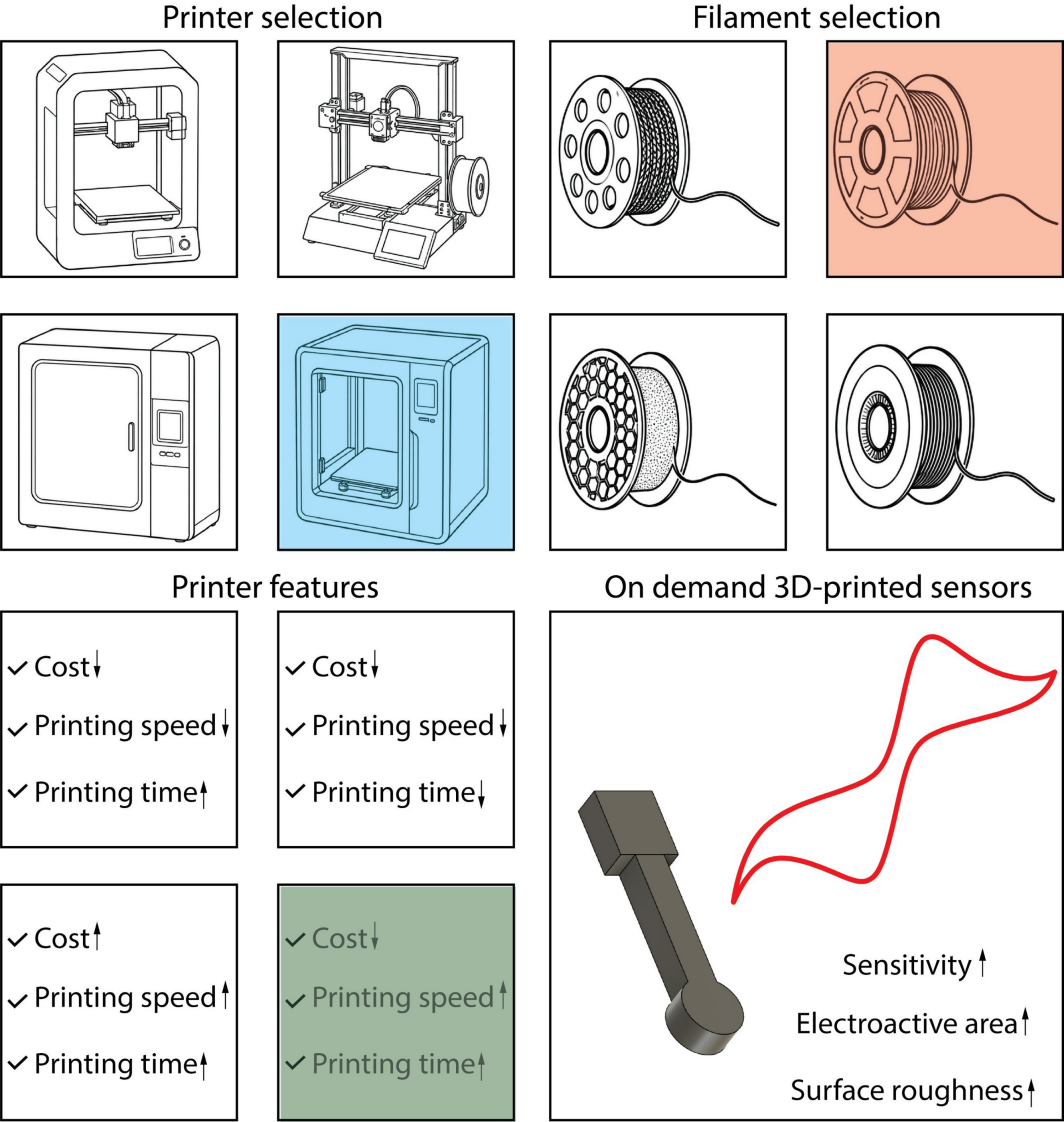
Workflow illustrating the selection and evaluation of FDM printing platforms for electrochemical sensor fabrication, including printer class selection, conductive filament choice according to the targeted application, assessment of key printer characteristics such as cost, printing speed, fabrication time, automation level, and hardware configuration, followed by the evaluation of the resulting electrode morphology and electrochemical performance.

The aim of this study is to investigate the influence of FDM printer classes and hardware configurations on the fabrication and performance of 3D‐printed electrochemical electrodes (Figure 1). Four representative platforms, spanning consumer‐level and industrial‐grade systems (Bambu Lab X1‐Carbon, Bambu Lab A1 Mini, Creality Ender‐3 V2 Neo, and FlashForge Creator 4‐HS), were evaluated to balance key trade‐offs in equipment cost, printing speed, fabrication time, automation, and hardware flexibility. Conductive PLA was selected as a reference material to enable a consistent comparison across platforms, while alternative conductive filaments, such as TPU‐based composites [7, 31], can be selected depending on the targeted application and desired electrode properties. As summarized in Figure 1, printer characteristics, including cost, printing speed, fabrication time, motion control, and extrusion stability, directly govern electrode morphology and electrochemical behavior. These effects were systematically assessed through surface roughness, ECSA, and electrochemical response evaluations, establishing a practical framework for selecting reliable, cost‐effective fabrication, and scalable sensor manufacturing platforms.

### 3.1 Morphological Characterization of Electrodes by SEM

**FIGURE 2:**
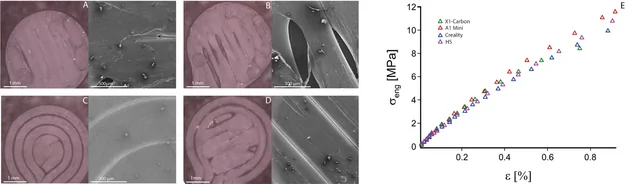
Morphological characterization of 3D‐printed electrodes fabricated by the four FDM printing platforms. Optical images (left) and SEM micrographs (right) of the X1‐Carbon (A), A1 Mini (B), Creality (C), and HS (D). Engineering stress–strain curves obtained at different stretching rates for all samples as indicated in the legend. The applied stretching rate is 1 s^−1^ (E).

SEM analysis was performed to investigate the morphology of the 3D‐printed electrode architectures and to assess the structural features resulting from the additive manufacturing process. Representative micrographs of all samples investigated are shown in Figure 2.

All samples exhibited well‐defined architectures and the characteristic structural features associated with the 3D‐printing process. At low magnification, the overall geometry of the electrodes and the organization of the printed filaments were clearly observed.

In detail, some differences were mainly observed in the overall electrode design and filament arrangement. While A1 Mini and X1‐Carbon displayed similar architectures characterized by elongated internal openings and a predominantly linear filament organization, Creality and HS exhibited a more concentric filament arrangement, reflecting a different printing path and electrode geometry. Higher‐magnification SEM images revealed comparable surface morphologies among the investigated samples, with clearly distinguishable printed tracks and preserved filament integrity. Overall, the SEM analysis confirmed the successful fabrication of all electrode architectures while highlighting differences primarily related to their geometrical design and filament organization. Additionally, the elemental composition of the 3D‐printed carbon electrodes was evaluated by EDX analysis. As shown in Figure S2, the EDX spectra consistently identified carbon and oxygen as the predominant elements in all investigated samples, confirming the carbon‐based composition of the printed electrodes. The presence of these elements is in accordance with other reported studies on conductive PLA filaments [32]. Elemental mapping revealed homogeneous distribution of both elements across the analyzed surfaces. Minor amounts of additional elements (e.g., Na and Cl) were sporadically detected and are likely associated with residual impurities from the fabrication process or sample handling. Since the same commercial conductive PLA filament was used for all printing platforms, substantial differences in elemental composition were not expected. The comparable C and O composition observed across the electrodes therefore confirms the consistency of the starting material while suggesting that the differences in electrochemical behavior are more likely associated with printing‐dependent characteristics, including surface morphology, fila‍ment organization, and accessibility of the conductive network, rather than variations in elemental composition.

### 3.2 Tensile Analysis

**FIGURE 3:**
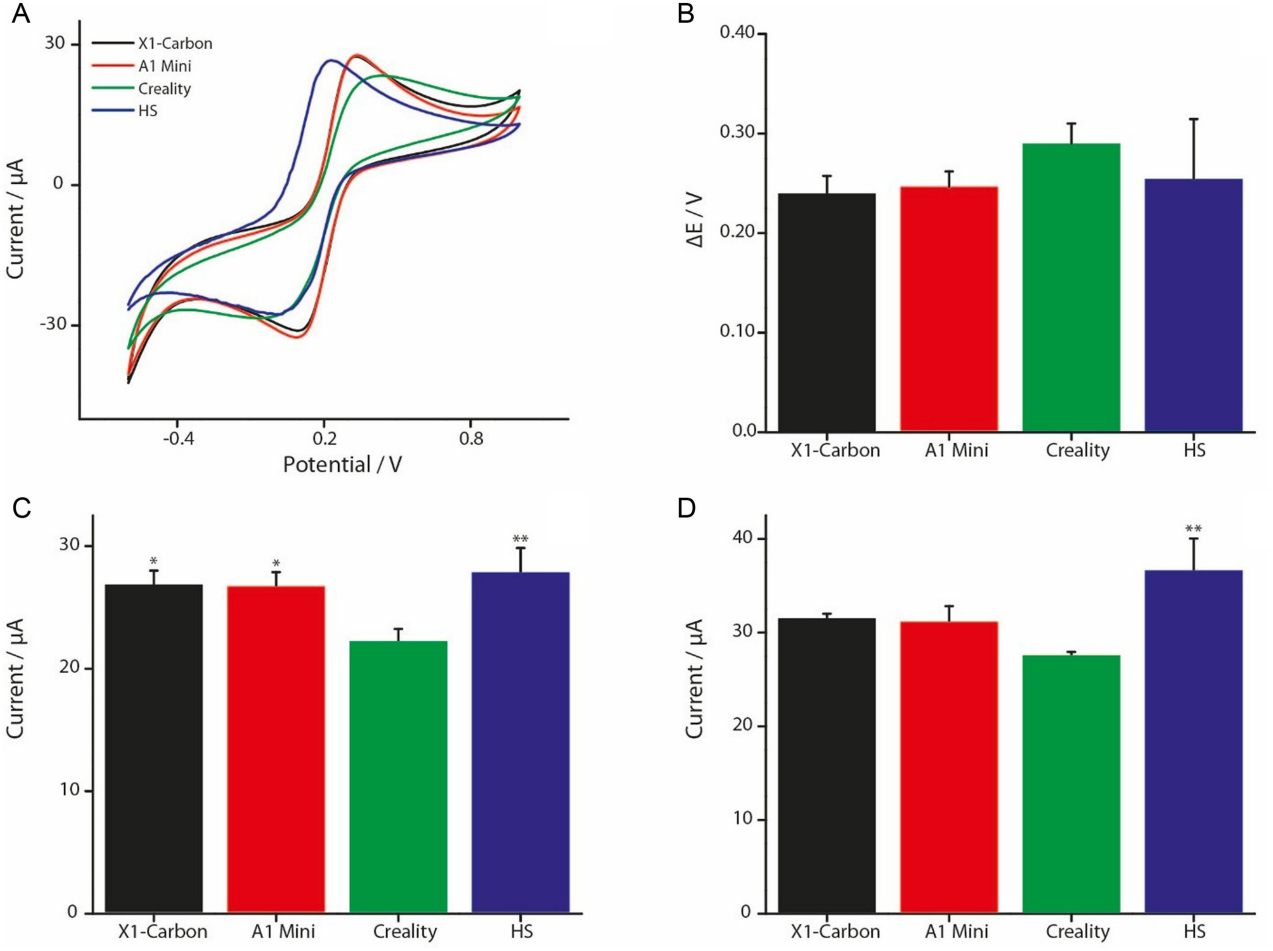
Electrochemical characterization of 3D‐printed electrodes using 1 mM potassium ferricyanide as a redox probe. (A) Representative cyclic voltammograms recorded at 0.1 V s^−1^; (B) peak‐to‐peak separation (Δ*E*); (C) anodic peak current (*I*_pa_); and (D) cathodic peak current (*I*_pc_). Data are expressed as mean ± SD (*n* = 3). Statistical significance was evaluated by one‐way ANOVA followed by Tukey’s multiple comparisons test. *p < 0.05, **p < 0.01 versus Creality.

Although mechanical characterization was secondary to the electrochemical evaluation, it was included to assess the structural integrity of the printed electrodes during handling and integration into the final device. The 3D‐printed electrodes are mechanically handled and usually incorporated into click‐in mechanisms for a final 3D‐printed device, making adequate mechanical integrity important for reliable assembly and operation [4, 33]. Tensile testing here also provides complementary information on whether printer‐dependent differences in motion control, filament deposition, and layer formation influence the mechanical properties of the resulting structures. The tensile analysis was performed to understand how different FDM printing platforms influence the mechanical integrity of the fabricated structures. Figure 3 reports the tensile stress–strain curves obtained at a stretching rate of 1 s^−1^.

The complete tensile stress–strain curves obtained at stretching rates of 2.12 × 10^−3^, 4.64 × 10^−3^, and 1.0 × 10^−^^2^ s^−1^ for all investigated samples are presented in Figure S3. Owing to the maximum normal force limitation of the rheometer, combined with the relatively large cross‐sectional area of the printed specimens, the quantitative analysis was restricted to the linear elastic regime, corresponding to strains between 0% and 0.4%. Within this range, Young’s modulus could be reliably determined. The experimental results indicate that all specimens exhibit similar linear elastic response. Despite the different preparation and printing equipment adopted for the investigated materials, no significant differences were observed within the elastic regime. Consequently, all specimens yielded comparable values of Young’s modulus, with an average value of 1.40 ± 0.13 GPa. The measured Young’s modulus is consistent with values typically reported for comparable polymeric materials and falls within the variability generally observed in tensile testing according to ASTM D638‐22, the internationally recognized standard that specifies the procedures for measuring tensile properties [34]. It should be considered that polyester sheets, used as the gold standard for screen‐printing, displayed a maximum stress of ca. 147 MPa [35].

Differences among the samples become apparent only at larger strains, where deviations from the linear elastic behavior are observed. As highlighted by the SEM micrographs, the specimens exhibit different internal morphologies resulting from variations in the degree of filling achieved during the 3D‐printing process. Differences in void content and pore distribution are expected to be responsible for the observed divergence in the stress–strain curves within the nonlinear regime while leaving the elastic response substantially unaffected. It should be noted that, due to the instrumental force limitation, the present quantitative analysis was restricted to the linear elastic regime (0%–0.4% strain). Therefore, mechanical properties at larger deformation, such as failure behavior, flexural performance, and interlayer adhesion, were not quantitatively assessed and could be further investigated in future studies. In applications requiring greater flexibility, alternative printing materials, such as TPU‐based filaments [36], could also be considered.

### 3.3 Electrochemical Characterization of the 3D‐Printed Electrodes

To assess the influence of the 3D‐printing approach on the electrochemical properties of the fabricated electrodes, different printing platforms were evaluated under their respective operating conditions. The electrode geometry, conductive filament, infill pattern and density, nozzle temperature, bed temperature, and postprinting electrochemical pretreatment were kept constant across all platforms, while printing parameters related to motion control and material deposition, including printing speed, acceleration, first‐layer speed, infill speed, and wall speed, were set according to the default or recommended configurations of the respective printer/slicer systems (Table S1). This approach was adopted to evaluate the practical capabilities of each 3D‐printing platform under its representative operating conditions without individually optimizing the printing parameters for electrochemical performance. Such parameters can affect filament deposition, layer formation, surface morphology, and the distribution of conductive pathways within the printed structure, ultimately influencing the electrochemical behavior of the electrodes. Thus, the comparison presented below reflects the performance of the complete 3D‐printing fabrication systems, encompassing both platform‐specific characteristics and their associated printing conditions.

### 3.4 Electrochemical Characterization of the 3D‐Printed Electrodes Using Potassium Ferricyanide

The electrochemical performance of the 3D‐printed electrodes was evaluated using 1 mM potassium ferricyanide in 1 M KCl as a standard redox probe in the potential window between −0.6 and +1.0 V (vs. Ag/AgCl). As an inner‐sphere redox mediator [37], [Fe(CN)_6_]^3−^ is influenced by the surface chemistry, defects, and functional groups, making it a useful probe for assessing the overall electrochemical activity of the electrode surface. In the present conductive PLA electrodes, it was therefore considered a complementary probe reflecting both surface and interfacial characteristics rather than a selective measure of surface chemistry alone. CV experiments were performed at scan rates ranging from 0.01 to 0.2 V s^−1^ to investigate charge transfer kinetics and mass transport behavior at the electrode/electrolyte interface (Figure S4 and RSD% Table S2). Key electrochemical parameters are summarized in Table 1, and representative cyclic voltammograms along with the extracted analytical parameters are shown in Figure 3.

**TABLE 1:** Electrochemical performance parameters obtained from CV analysis of potassium ferricyanide at different 3D‐printed carbon electrodes.

Potassium ferricyanide	X1‐Carbon	A1 Mini	Creality	HS
Δ*E*, V	0.24 ± 0.02	0.25 ± 0.02	0.29 ± 0.02	0.25 ± 0.06
Electroactive area, cm^2^	0.16 ± 0.01	0.16 ± 0.01	0.13 ± 0.01	0.17 ± 0.01
Roughness factor	0.32 ± 0.01	0.32 ± 0.02	0.26 ± 0.01	0.33 ± 0.03
Anodic slope, µA/Vs^−1^	88	94	60	107
Slope ratio	0.93	1.00	1.03	1.37

All electrodes exhibited quasireversible electrochemical behavior, as indicated by peak‐to‐peak separation (Δ*E*) values significantly exceeding the theoretical Nernstian limit of 59 mV for a one‐electron reversible process. The Δ*E* values ranged from 0.24 V for the X1‐Carbon to 0.29 V for the Creality electrode, confirming quasireversible behavior across all printing platforms. Such responses are characteristic of FDM 3D‐printed electrodes fabricated from conductive thermoplastic composites and can arise from the combined effects of the limited electrical conductivity of the carbon‐filled polymer matrix, surface structure, conductive pathway distribution, and interfacial properties [38]. The dispersion of conductive carbon particles within the insulating PLA matrix increases the internal and contact resistance of the electrode, leading to Ohmic potential losses that broaden the peak separation. Therefore, the observed Δ*E* values are more likely a consequence of the intrinsic resistive nature of the printed composite than of inherently slow heterogeneous electron‐transfer kinetics. Nonetheless, the lower Δ*E* exhibited by the X1‐Carbon is consistent with a more favorable overall electrochemical response and may indicate lower contributions from resistive and interfacial limitations under the conditions employed. The ECSA varied only slightly among the electrodes (0.13–0.17 cm^2^), indicating that factors other than electroactive area, such as surface morphology, conductive pathway distribution, and electrical resistance, are likely to play a more significant role in the observed electrochemical differences. In contrast, the roughness factor showed noticeable variation, with HS exhibiting the highest value (0.33), suggesting greater surface heterogeneity likely induced by the printing process. The anodic peak current (*I*_pa_) ranged from 22 ± 1 μA (Creality) to 28 ± 2 μA (HS), while the cathodic peak current (*I*_pc_) ranged from 28 ± 1 μA (Creality) to 37 ± 4 μA (HS). The systematically higher *I*_pc_ compared to *I*_pa_ across all platforms is consistent with the quasireversible nature of the redox process.

The anodic‐to‐cathodic slope ratio indicates the symmetry of the redox process, with values closer to unity reflecting a more balanced and ideal electrochemical response. The slope ratio varied between 0.926 and 1.37 across the electrodes, with values nearest to 1 indicating more symmetric oxidation and reduction processes. In contrast, larger deviations from unity suggest asymmetry in the redox response, which may arise from differences in mass transport, electroactive surface accessibility, or resistive effects associated with the printed electrode structure.

Overall, the HS printer exhibited the highest anodic slope, together with the largest ECSA and roughness factor, indicating enhanced electroactive accessibility and a greater density of available active sites. In contrast, the Creality printer showed the largest peak‐to‐peak separation, the lowest ECSA and roughness factor, and the smallest anodic slope, suggesting reduced electrochemical activity and higher resistive losses. The X1‐Carbon exhibited the lowest Δ*E*, corresponding to the smallest peak separation under the experimental conditions, while maintaining an ECSA and roughness factor comparable to those of the A1 Mini and HS. Interestingly, although the A1 Mini displayed a slightly higher Δ*E* than the X1‐Carbon, its anodic‐to‐cathodic slope ratio was closest to unity, indicating the most symmetric redox response and behavior closest to an ideal diffusion‐controlled process. Collectively, these findings demonstrate that the choice of 3D‐printing fabrication system, together with its associated printing conditions, is associated with differences in the electrochemical properties of the fabricated electrodes. These differences involve not only peak separation but also the electroactive surface properties and redox symmetry, highlighting the importance of considering the complete printing system when evaluating and selecting a platform for electrochemical sensor fabrication.

### 3.5 Electrochemical Characterization of the 3D‐Printed Electrodes Using Hexammine Ruthenium (III)

**FIGURE 4:**
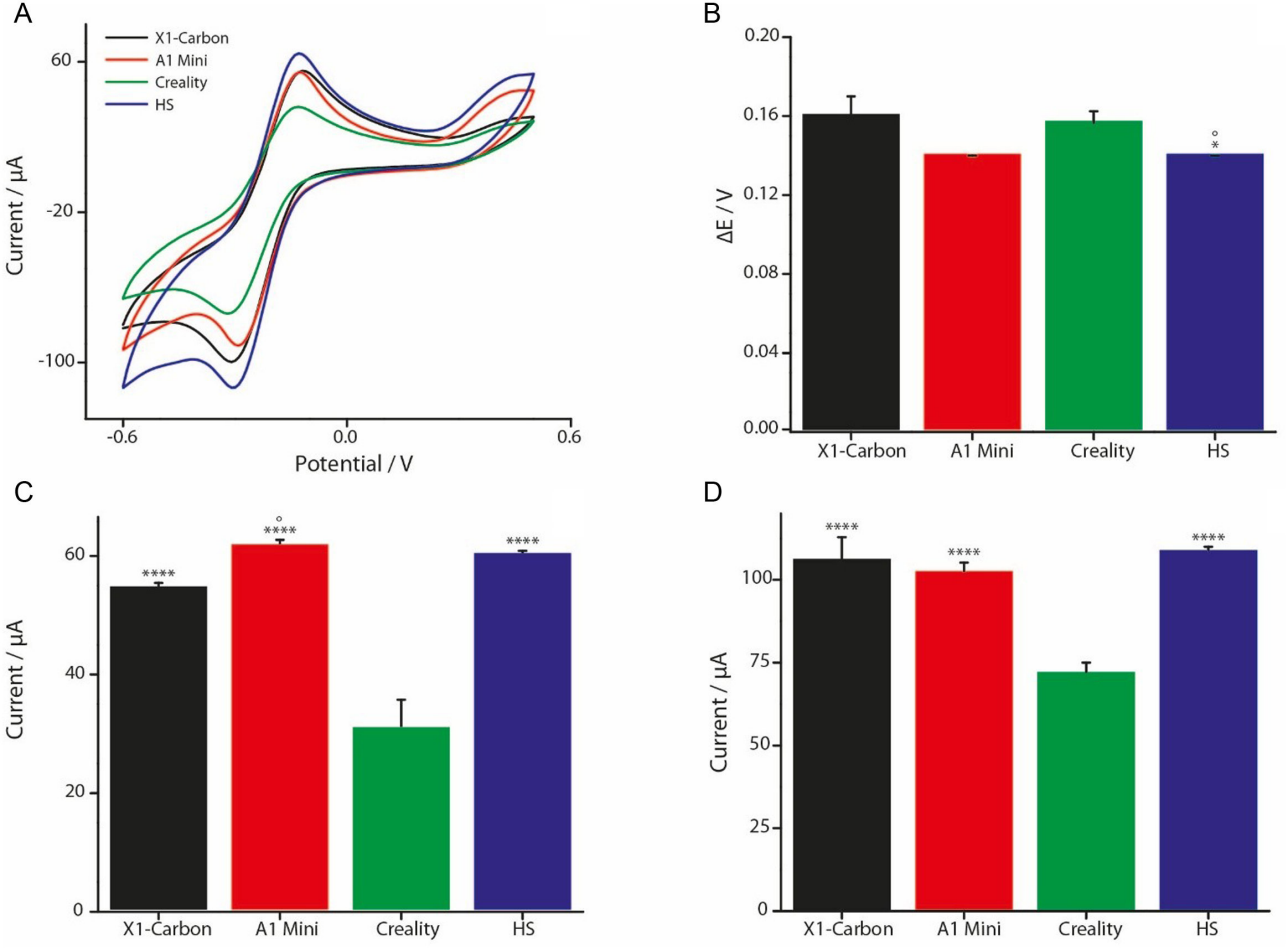
Electrochemical characterization of 3D‐printed electrodes using 1 mM hexammine ruthenium (III) chloride as a redox probe. (A) Representative cyclic voltammograms recorded at 0.1 V s^−1^; (B) peak‐to‐peak separation (Δ*E*); (C) anodic peak current (*I*_pa_); and (D) cathodic peak current (*I*_pc_). Data are expressed as mean ± SD (*n* = 3). Statistical significance was evaluated by one‐way ANOVA followed by Tukey’s multiple comparisons test. *p < 0.05 and ****p < 0.0001 versus Creality; °*p *< 0.05 versus X1‐Carbon.

The electrochemical behavior of the 3D‐printed electrodes was further investigated using 1 mM hexammine ruthenium (III) chloride in 1 M KCl. [Ru(NH_3_)_6_]Cl_3_ was used as a redox probe in the potential window between −0.6 and +0.5 V (vs. Ag/AgCl). As an outer‐sphere redox mediator [39], [Ru(NH_3_)_6_]^3+^ is comparatively less influenced by surface chemistry interactions and provides complementary information on electroactive accessibility and charge‐transfer behavior. However, although outer‐sphere electron‐transfer processes are generally less dependent on specific surface chemical interactions, the measured response can still be influenced by the interfacial environment, including surface charge, ionic strength, carbon structure, and the accessibility and conductivity of the printed composite. Therefore, the ruthenium probe should not be considered completely independent of the electrode surface but rather as a complementary probe that is comparatively less affected by specific surface chemical interactions. CV experiments were performed at scan rates ranging from 0.01 to 0.2 V s^−1^ (Figure S5 and RSD% Table S2). Key electrochemical parameters are summarized in Table 2, and representative cyclic voltammograms along with the extracted analytical parameters are shown in Figure 4.

**TABLE 2:** Electrochemical performance parameters obtained from CV analysis of hexaammineruthenium chloride at different 3D‐printed carbon electrodes.

Hexaamminoruthenium	X1‐Carbon	A1 Mini	Creality	HS
Δ*E*, V	0.16 ± 0.01	0.140 ± 0.006	0.16 ± 5.8 × 10^−3^	0.140 ± 0.003
Electroactive area, cm^2^	0.31 ± 0.01	0.35 ± 0.01	0.17 ± 0.03	0.34 ± 0.01
Roughness factor	0.61 ± 0.01	0.69 ± 0.01	0.34 ± 0.06	0.67 ± 0.01
Anodic slope, µA/Vs^−1^	202	238	133	192
Slope ratio	0.99	0.99	1.12	1.06

All electrodes, also in this study, exhibited quasireversible behavior, with Δ*E* values ranging from 0.140 V (HS and A1 Mini) to 0.160 V (X1‐Carbon), notably lower than those observed with potassium ferricyanide, indicating smaller peak separations under the experimental conditions. Although this behavior is consistent with the generally favorable charge‐transfer characteristics of outer‐sphere redox processes, Δ*E* should not be interpreted exclusively as a measure of electron‐transfer kinetics as it can also reflect uncompensated resistance, surface heterogeneity, conductive‐site distribution, and interfacial effects. The anodic peak current (*I*_pa_) ranged from 31 ± 5 μA (Creality) to 62 ± 1 μA (A1 Mini), while the cathodic peak current (*I*_pc_) ranged from 73 ± 1 μA (Creality) to 109 ± 1 μA (HS). As observed with potassium ferricyanide, *I*_pc_ values were systematically higher than *I*_pa_ across all platforms, and both peak currents were substantially larger than those recorded with potassium ferricyanide, consistent with the higher anodic slope values. The ECSA showed greater variability compared with the potassium ferricyanide results, ranging from 0.173 cm^2^ (Creality) to 0.346 cm^2^ (A1 Mini), with a corresponding trend in the roughness factor (0.344–0.687). The anodic‐to‐cathodic slope ratios were closer to unity for most electrodes (0.99–1.06), except for the Creality (1.12), indicating more symmetric redox behavior compared to potassium ferricyanide results. The Bambu Lab A1 Mini exhibited the highest anodic slope (238.5 μA Vs^−1^), suggesting the greatest electroactive accessibility among the tested electrodes.

Overall, the A1 Mini exhibited the most favorable electrochemical performance, combining a low peak‐to‐peak separation, the highest ECSA, the largest roughness factor, and the highest anodic slope, indicating enhanced electroactive accessibility and efficient charge transport. The HS displayed the lowest Δ*E*, indicative of the most reversible electrochemical response, while also exhibiting ECSA and roughness factor values comparable to those of the A1 Mini. In contrast, Creality showed a substantially lower ECSA, the smallest roughness factor, and the lowest anodic slope, suggesting reduced electroactive accessibility and less efficient charge transport. The X1‐Carbon exhibited intermediate behavior, with electrochemical parameters generally comparable to those of the HS, although with a slightly larger Δ*E* and lower anodic slope. Furthermore, the anodic‐to‐cathodic slope ratios remained close to unity for the X1‐Carbon (0.99), A1 Mini (0.99), and HS (1.06), indicating highly symmetric redox behavior, whereas the larger deviation observed for the Creality electrode (1.12) suggests a less balanced oxidation and reduction response.

### 3.6 The Impact of the Polymer Matrix on Roughness Factors

As previously reported in the literature, raw 3D‐printed carbon electrodes fabricated from commercial conductive PLA composites typically require a postprinting chemical activation step using sodium hydroxide (NaOH) to become electrochemically active [40, 41, 42] due to the well‐established “polymer masking effect” associated with the FDM process. During extrusion and deposition, the thermoplastic binder preferentially accumulates at the outer surface of the printed structure, generating a polymer‐rich skin layer that covers the embedded conductive carbon network, resulting in poor electrochemical performance of the as‐printed electrode.

For Protopasta Conductive PLA, this phenomenon is particularly significant because the manufacturer reports a filament composition of greater than 67 wt.% polylactide resin, less than 20 wt.% conductive carbon black, and less than 13 wt.% additional constituents. This composition ensures printability and mechanical stability but limits the electrochemically active surface. Because carbon black is only a minor component, much of the electrode surface remains covered by insulating polymer, leaving only electrolyte‐exposed, electrically connected carbon particles available for electron‐transfer reactions [25].

NaOH treatment selectively hydrolyzes and removes the superficial polymer‐rich layer, exposing previously buried carbon black particles and increasing the number of conductive pathways accessible to the electrolyte. However, it does not convert the entire geometric surface into conductive carbon; instead, it creates a heterogeneous interface composed of exposed carbon‐rich regions interspersed with residual polymer domains. As a result, only a fraction of the nominal geometric area participates in electrochemical reactions [43]. ECSA lower than the geometric surface area are therefore expected and should be interpreted in the context of composite material architecture rather than as evidence of unsuccessful activation [25].

In conventional carbon or metallic electrodes, roughness factors greater than unity are frequently observed due to surface texturing and porosity, and microstructural features increase the electrochemically accessible area beyond the geometric area [44, 45]. Conductive PLA composites behave differently. Since the electroactive area is constrained by both surface morphology and the fraction of conductive material simultaneously exposed to the electrolyte and electrically connected to the bulk electrode, roughness factors below unity are expected. Variations in the roughness factor between printing platforms therefore reflect differences in filament deposition, layer fusion, and conductive network connectivity generated during the fabrication process. Although all electrodes were fabricated from the same conductive filament and subjected to the same chemical activation protocol, differences in extrusion dynamics and print quality can significantly influence the accessibility of the conductive carbon network. Higher roughness factors indicate a greater electrochemically accessible carbon after activation, whereas lower values suggest a larger insulating polymer‐dominated region. Therefore, the roughness factor serves as an indirect assessment of how effectively each printing platform preserves and exposes the conductive carbon network within the composite electrode.

### 3.7 Inner‐Sphere Versus Outer‐Sphere Probe Behavior

The comparison between potassium ferricyanide and hexaammineruthenium chloride revealed differences in their electrochemical responses across all printing platforms. The roughness factor values obtained using potassium ferricyanide were consistently lower (0.26–0.33) than those measured with hexaammineruthenium (0.34–0.69), highlighting differences in the electrochemical response of the two probes. Potassium ferricyanide is commonly classified as an inner‐sphere redox probe, and its response can be influenced by specific surface interactions, including surface functional groups, defects, and the accessibility of carbon‐rich regions. However, its electrochemical behavior is not determined exclusively by these interactions and can also be affected by surface charge, carbon microstructure, ionic strength, and the interfacial environment. In contrast, hexaammineruthenium(III) undergoes an outer‐sphere electron‐transfer process and is comparatively less dependent on specific surface chemical interactions, providing complementary information on electroactive accessibility and charge‐transfer behavior. Thus, the differences observed between the two probes should be interpreted as the combined result of their distinct electron‐transfer mechanisms and the physicochemical characteristics of the printed conductive PLA electrodes.

Collectively, the A1 Mini and HS demonstrated the most favorable overall electrochemical characteristics across both probes. X1‐Carbon also exhibited competitive performance, particularly in terms of lower peak separation with potassium ferricyanide, which indicates a smaller ΔEp under the present experimental conditions but cannot by itself be attributed exclusively to reduced Ohmic losses or faster electron‐transfer kinetics. In contrast, Creality generally showed lower electroactive area, roughness factor, and current responses, suggesting differences in the accessibility and formation of the conductive network during printing.

To further investigate whether differences in the printing process affected the physical continuity of the deposited material, the surface material coverage was quantitatively assessed from SEM images using ImageJ analysis (Table S3 and Figure S6). The X1‐Carbon exhibited the highest surface coverage (94.11%), followed by the A1 Mini (90.00%), HS (86.95%), and Creality (76.64%). This analysis confirmed that the different 3D‐printing platforms produced electrodes with substantially different degrees of material deposition and surface continuity. Importantly, the observed differences in coverage provide additional morphological context for the electrochemical results as variations in the continuity of the deposited conductive material can influence the effective surface accessible to the electrolyte and, consequently, the measured current response. The lower and more variable surface coverage observed for the Creality‐printed electrodes is consistent with their generally lower electrochemical performance, whereas the higher coverage values observed for the other platforms were associated with higher overall electrochemical responses. Nevertheless, surface coverage alone does not fully determine electrode performance as the response also depends on factors such as surface chemistry, conductive‐network formation, roughness, and probe‐specific electron‐transfer behavior.

Taken together, the complementary redox‐probe measurements and the quantitative SEM analysis indicate that the electrochemical differences among the printing platforms arise from multiple, interrelated factors rather than from a single surface property. Ferricyanide provides a response influenced by surface and interfacial characteristics, whereas hexaammineruthenium(III) is comparatively less dependent on specific surface chemistry and provides complementary information on electroactive accessibility and charge‐transfer behavior. The surface coverage analysis further demonstrates that the printing platform affects the continuity and extent of material deposition, which may contribute to differences in electrochemical accessibility. Because neither redox probe nor morphological coverage alone selectively reports a single electrode property, their combined use provides a more comprehensive assessment of how the printing process influences the structural and electrochemical characteristics of the conductive PLA electrodes.

### 3.8 Cost Considerations

A cost analysis was performed to evaluate the economic feasibility of electrode fabrication across the four printing platforms based on both the material consumption estimated by the slicer software and the experimentally measured electrode and material waste mass. The analysis presented in this section focuses specifically on the sensor fabrication cost and associated material consumption/waste rather than providing a complete total cost of ownership analysis. The conductive PLA filament (Protopasta, Vancouver, WA, USA) was purchased at 61.5 € per 500 g, corresponding to a unit cost of 0.123 € g^−1^. The acquisition cost of the four printing platforms varied substantially, ranging from approximately €185–300 for the Bambu Lab A1 Mini and €250–330 for the Creality Ender‐3 V2 Neo to €1.1–1.3 K for the Bambu Lab X1‐Carbon and €9–12 K for the FlashForge Creator 4‐HS. These differences in initial investment represent an important practical consideration when selecting a printing platform and demonstrate that the material cost per electrode alone does not reflect the overall economic implications of printer selection.

The total filament consumption per print batch (10 electrodes) was calculated by accounting for both the material used for electrode fabrication and the filament consumed during nozzle cleaning and purge operations. The resulting material usage, as estimated by the slicer software and verified through the measured electrode mass, ranged from 1.00 g (Creality) to 1.41 g (X1‍‐‍Carbon), corresponding to a material cost per batch of 0.123 and 0.173 €, respectively. The A1 Mini and HS showed intermediate values of 1.17 g (0.144 €) and 1.19 g (0.146 €), respectively.

Despite these differences in total filament consumption, the experimentally measured masses of the individual electrodes were highly comparable (0.103–0.109 g), indicating that the variation in material consumption was primarily associated with printer‐dependent waste and auxiliary printing operations rather than differences in the amount of material constituting the final electrodes. This comparable electrode mass further indicates that the differences in electrochemical response are unlikely to be primarily driven by the amount of conductive PLA present in each electrode but rather by printing‐dependent differences in surface morphology, ECSA, and accessibility of the conductive network. Table 3 summarizes the fabrication cost and time requirements associated with electrode production using the four printing platforms.

**TABLE 3:** Cost and time analysis of electrode fabrication across the four printers.

Printers	Actual printing time (10 electrodes)	Printing setup time	Filament use, g	Cost per batch, €	Electrode weight, g	Cost per electrode, €
X1‐Carbon	4 min 42 s	8 min 13 s	1.41	0.17	0.1090 ± 3 × 10^−4^	0.013
A1 Mini	4 min 45 s	7 min 2 s	1.17	0.14	0.1070 ± 4 × 10^−4^	0.013
Creality	13 min 34 s	48 s	1.00	0.12	0.1030 ± 1 × 10^−4^	0.012
HS	20 min	0 s	1.19	0.15	0.1060 ± 4 × 10^−4^	0.013

Regarding fabrication time, the Bambu Lab printers demonstrated the shortest actual printing time (4 min 42 s and 4 min 45 s for the X1‐Carbon and A1 Mini, respectively), whereas the HS required the longest (20 min). However, when accounting for printing setup time, the total fabrication time changed considerably among platforms. Creality showed the shortest overall production time (14 min 22 s, including 13 min 34 s printing time and 48 s setup time), while X1‐Carbon required an additional 8 min 13 s of automated setup time, resulting in a longer total processing time despite its faster printing speed, while the cost per electrode was comparable.

To further contextualize the sustainability and practicality of the fabricated 3D‐printed electrodes, redox mediators were measured as a proof of concept, and the corresponding CACI, MoGAPI, AGSA‐Prep, and SAMI scores were calculated (Figure S7). Numerous such metrics exist, each offering a distinct lens, and selecting the most appropriate ones is important as they collectively highlight key advantages of decentralized analytical platforms, including on‐site applicability and minimal sample requirements [46, 47, 48, 49, 50, 51]. CACI (85/100) [52] highlighted strong portability and semiautomation, reflecting the decentralized, low‐cost fabrication workflow. MoGAPI (87/100) [53] confirmed the method’s overall greenness, driven by minimal reagent use and reduced material waste. AGSA‐Prep (95/100) [54] captured low energy consumption during sample preparation, while SAMI (81/100) [55] linked the platform to relevant UN Sustainable Development Goals. Notably, a growing number of complementary metrics and tools have been developed in recent years to assess the greenness, practicality, and sustainability of analytical procedures from multiple perspectives, each offering valuable and distinct insights. Together, these metrics provide a multidimensional benchmark of the platform’s sustainability and practicality.

## 4 Conclusion

The electrochemical, mechanical, and morphological characterization of conductive PLA electrodes fabricated using four 3D‍‐‍printers demonstrates that all systems can produce sensors with electrochemical functionality; however, the principal distinctions were reflected in electrochemical performance. The A1 Mini and HS produced electrodes with the largest ECSA and highest roughness factors, indicating greater accessibility of the conductive network after activation. Both platforms also exhibited good reproducibility as evidenced by the low variability between replicates. X1‐Carbon delivered similarly competitive performance and achieved the lowest peak‐to‐peak separation with potassium ferricyanide, suggesting efficient electron‐transfer kinetics. In contrast, the Creality printer yielded the lowest roughness factor (0.34), indicating limited electrochemical accessibility, together with the highest variation in peak current, reflecting reduced fabrication consistency. These differences are likely related to variations in filament deposition and layer consolidation, which influence the exposure of conductive fillers within the PLA matrix.

The use of complementary redox probes further demonstrated that printer performance depends on the electrochemical property evaluated. Potassium ferricyanide was more responsive to electron‐transfer characteristics and surface chemistry, whereas ruthenium provided a better assessment of the accessible conductive network. Consequently, no platform was superior across every metric. Because material consumption was virtually identical, with an estimated fabrication cost of approximately €0.013 per electrode, printer selection should instead consider operational aspects such as automation, printing speed, and ease of use. The Bambu Lab X1‐Carbon and A1 Mini offer highly automated workflows that minimize user intervention, making them particularly suitable for routine laboratory fabrication. HS combines high electrochemical performance with industrial‐oriented operation, whereas the Creality platform offers the greatest affordability and hardware flexibility but requires greater operator involvement and delivers lower reproducibility. Accordingly, there is no single universally superior 3D‐printing platform as the optimal choice depends on the specific requirements of the intended application. The A1 Mini represents a particularly attractive option when a combination of electrochemical performance, automation, and fabrication throughput is desired; the X1‐Carbon is advantageous when automation, speed, and a favorable electrochemical response are prioritized; HS is suited to applications requiring high electrochemical performance and industrial‐oriented operation, while Creality remains attractive for applications where low acquisition cost and hardware flexibility are particularly important.

These findings position FDM‐printed conductive PLA electrodes as accessible and cost‐effective platforms for electrochemical sensing applications, and the choice of printing platform should therefore be guided not only by electrochemical performance but also by practical considerations such as ease of use, laboratory footprint, fabrication throughput, and cost of ownership, in line with what has been previously highlighted in the literature [21].

## Author Contributions

**Gennaro Persico:** investigation, writing – original draft. **Panagiota M. Kalligosfyri:** conceptualization, methodology, investigation, validation, formal analysis, writing – original draft, review and editing, supervision. **Elena Lagreca:** validation, formal analysis. **Vincenzo Ianniello:** validation, formal analysis. **Concetta Di Natale:** validation, formal analysis. **Michela Volgare:** resources. **Antonio Cutolo**: resources. **Filippo Defina:** resources. **Waleed Alahmad:** writing – review and editing, **Abdulaziz K. Assaifan:** funding, supervision, writing – review and editing, **Mariarosaria Bucci:** writing – review and editing, supervision. **Stefano Cinti:** conceptualization, methodology, formal analysis, writing – review and editing, funding, supervision, project administration.

## Funding

This study was supported by the Royal Society of Chemistry (L25‐8786879940), Regione Campania (FSE+ 2021/2027), AIRC – MFAG 2022 project (27586), and King Saud University (ORF‐2026‐1314).

## Conflicts of Interest

The authors declare no conflicts of interest.

## Supporting information

Supplementary Material

## Data Availability

The data that support the findings of this study are available on request from the corresponding author. The data are not publicly available due to privacy or ethical restrictions.
