# Supplementary material for: Tuning the Properties of 3D‐Printed Electrochemical Devices by Using Different Fused Deposition Modeling Printers and Setups
Source: ChemistryOpen. 2026 Sep 24;15(10):e70303. doi: 10.1002/open.70303 (PMC13602825; doi:10.1002/open.70303)
Supplement: Supplementary file 1 [file OPEN-15-e70303-s001.pdf]

# Supporting Information

# Tuning the Properties of 3D-Printed Electrochemical Devices by Using Different FDM Printers and Setups

Gennaro Persico[a,§], Panagiota M. Kalligosfyri[a,§,*], Elena Lagreca[b], Vincenzo Ianniello[b], Concetta Di Natale[b], Michela Volgare[c], Antonio Cutolo[c], Filippo Defina[c], Waleed Alahmad[d], Abdulaziz K. Assaifan[e], Mariarosaria Bucci[a], Stefano Cinti[a,d,f,g,*]

[a] G. Persico, P. M. Kalligosfyri, M. Bucci, S. Cinti
University of Naples Federico II, Department of Pharmacy
Via D. Montesano 49, Naples 80131, Italy
E-mail: panagiota.kalligosfyri@unina.it; stefano.cinti@unina.it

[b] E. Lagreca, V. Ianniello, C. Di Natale
University of Naples Federico II, Dipartimento di Ingegneria Chimica, dei Materiali e della Produzione Industriale
P.le Tecchio 80, I-80125 Naples, Italy

[c] M. Volgare, A. Cutolo, F. Defina
3DnA s.r.l.
Viale Impero, 80038 Naples, Italy

[d] W. Alahmad, S. Cinti
Department of Chemistry, Faculty of Science, Chulalongkorn University
Bangkok 10330, Thailand

[e] A. K. Assaifan
Biomedical Technology Department, College of Applied Medical Sciences, King Saud University
Riyadh 12372, Saudi Arabia
E-mail: aassaifan@ksu.edu.sa

[f] S. Cinti
Sbarro Institute for Cancer Research and Molecular Medicine, Center for Biotechnology, College of Science and Technology, Temple University
Philadelphia, PA 19122, USA

[g] S. Cinti
Bioelectronics Task Force at University of Naples Federico II
Naples 80126, Italy

§These authors contributed equally.

## Table of content

Material and methods — Page S1

Table S1. Printing parameters and specifications of 3D printers used in this study — PageS2

Dimensions and configuration of the 3D-printed electrochemical cell — Page S3

Figure S2. EDX Elemental analysis — Page S3

Figure S3. Tensile testing of the 3D-printed — Page S4

Figure S4. Electrochemical characterization using potassium ferricyanide — Page S5

Figure S5. Electrochemical characterization using hexammine ruthenium (III) — Page S6

Figure S6. Quantitative analysis of surface material coverage from SEM Images — Page S9

Figure S7. Sustainability, practicality, and greenness of the 3D-printed electrodes — Page S9

## Material and methods

*Reagents and equipment*

Hexaammine ruthenium (III) Chloride ($[Ru(NH_3)_6]Cl_3$), potassium hexacyanoferrate (III) ($K_3[Fe(CN)_6]$), and potassium chloride (KCl) were purchased from Merck Life Science (St. Louis, MO, USA). All solutions were prepared in Milli-Q water produced in-house to 18 MΩ/cm, using a Milli-Q EQ 7015 Ultrapure Water Purification System (Darmstadt, Germany). Conductive inks (Ag/AgCl and graphite) were purchased from Sun Chemicals (USA). Flexible polyester film (HT5 Autostat) was kindly provided by McDermid Alpha (UK) and MacDermid Performance Solutions Italiana (Italy). The 3D-printed electrodes were printed using the Bambulab X1-Carbon (Shenzhen Tuozhu Technology Co., Ltd., Shenzhen, China), Bambulab A1 Mini (Shenzhen Tuozhu Technology Co., Ltd., Shenzhen, China), Creality Ender-3 V2 Neo (Shenzhen Creality 3D Technology, Shenzhen, China), FlashForge Creator 4-HS (Zhejiang Flashforge 3D Technology Co., Ltd., Jinhua, China). Protopasta conductive PLA filament 1.75 mm (Protoplant, Vancouver, WA, USA) was used to fabricate the 3D-printed electrodes. Electrochemical measurements were performed using the Emstat4S potentiostat (PalmSens, Houten, The Netherlands) connected to the PSTrace 5.11 software.

*Evaluation of electrodes' mechanical properties via tensile analysis*

To evaluate the mechanical properties of the 3D-printed specimens in the solid state, tensile tests were performed using an MCR 703 rheometer (Anton Paar, Graz, Austria) equipped with a linear motor and a tensile testing fixture. To minimize slippage between the specimens and the clamps during testing, P150 sandpaper was attached to the gripping surfaces in order to increase the interfacial friction and effective contact area. Furthermore, the clamps were tightened using a torque-limiting screwdriver set to a constant tightening torque of 50 cN·m, ensuring reproducible gripping conditions for all measurements. All tests were conducted at room temperature and at a constant stretching rate. During the experiments, the rheometer recorded the normal force and the distance between the clamps. Engineering stress was subsequently calculated from the measured force and the initial cross-sectional area of the specimens, while engineering strain was determined from the travelling distance.

*Scanning Electron Microscopy (SEM)*

The surface morphology of the 3D-printed electrodes was investigated using a Thermo Scientific™ Phenom ProX G6 desktop scanning electron microscope (Thermo Fisher Scientific, USA) equipped with a Secondary Electron Detector (SED). The electrodes were directly mounted on aluminum stubs and analyzed under high-vacuum conditions. SEM micrographs were acquired at an accelerating voltage of 5 kV and at different magnifications to evaluate the surface topography, layer-by-layer deposition pattern, and structural integrity of the printed electrodes. Digital images were collected using the integrated acquisition software, and representative micrographs from different regions of each electrode were selected for analysis.

*EDX Elemental Analysis*

The elemental composition of the 3D-printed carbon electrodes was characterized by energy-dispersive X-ray spectroscopy (EDX) coupled SEM. Before analysis, the samples were mounted on aluminum stubs using conductive carbon tape. Due to the intrinsic electrical conductivity of the carbon electrodes, no conductive metal coating was applied. SEM-EDX measurements were performed using a Phenom ProX G6 desktop scanning electron microscope (Thermo Fisher Scientific, Eindhoven, The Netherlands) equipped with an integrated silicon drift detector (SDD). Analyses were carried out under high-vacuum conditions using an accelerating voltage of 15 kV. EDX spectra and elemental maps were acquired from representative regions of each electrode to evaluate the elemental composition and its spatial distribution. Semi-quantitative elemental analysis was performed using the instrument software after automatic background subtraction and ZAF matrix correction. The elemental composition was expressed as both weight percentage (wt%) and atomic percentage (at%).

### 3D Printing parameters and experimental set up

**Table S1.** Printing parameters and specifications of the different 3D printers used in this study.

| Printer | Cost (€) | Printing Speed (mm/s) | Acceleration ($mm/s^2$) | First layer speed (mm/s) | Infill Speed (mm/s) | External Wall speed (mm/s) | Top Layer Speed (mm/s) |
|---|---|---|---|---|---|---|---|
| X1-Carbon | 1.100–1.350 | 200 | 10000 | 49 | 78 | 78 | 107 |
| A1 Mini | 185–300 | 200 | 6000 | 45 | 90 | 90 | 149 |
| Creality | 250–330 | 70 | 500 | 15 | 37 | 26 | 29 |
| HS | 9.000–12.000 | 70 | 1000 | 40 | 70 | 75 | 70 |

## Dimensions and configuration of the 3D-printed electrochemical cell

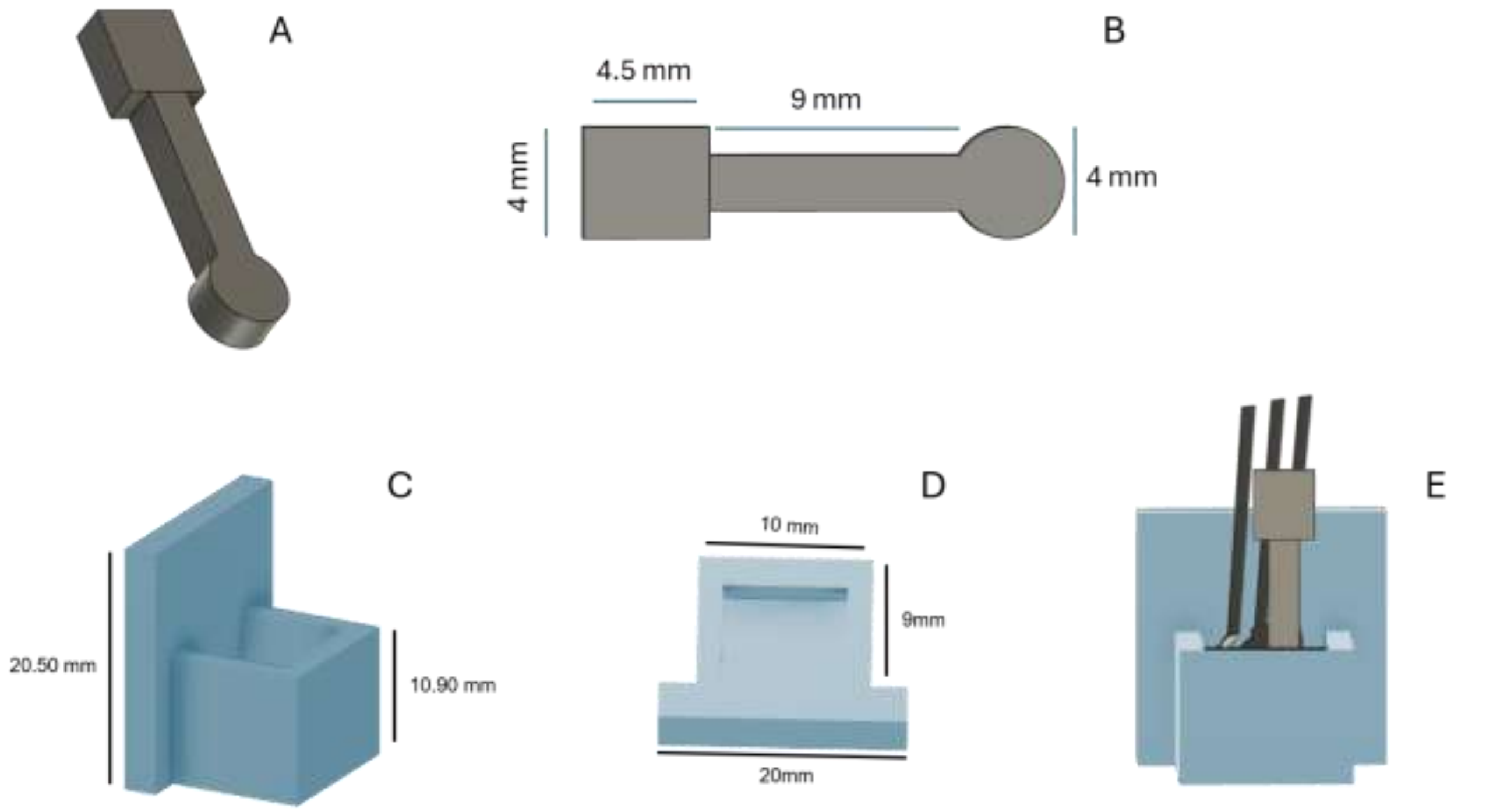


**Figure S1.** Electrode design and electrochemical cell configuration. (A) Representative 3D-printed conductive PLA electrode; (B) electrode dimensions; (C) lateral view of the electrochemical cell; (D) top view of the electrochemical cell; (E) schematic representation of the analytical configuration, showing the electrochemical cell assembled with the screen-printed reference and counter electrodes and the 3D-printed working electrode

## Supplementary Results

### *EDX Elemental Analysis*

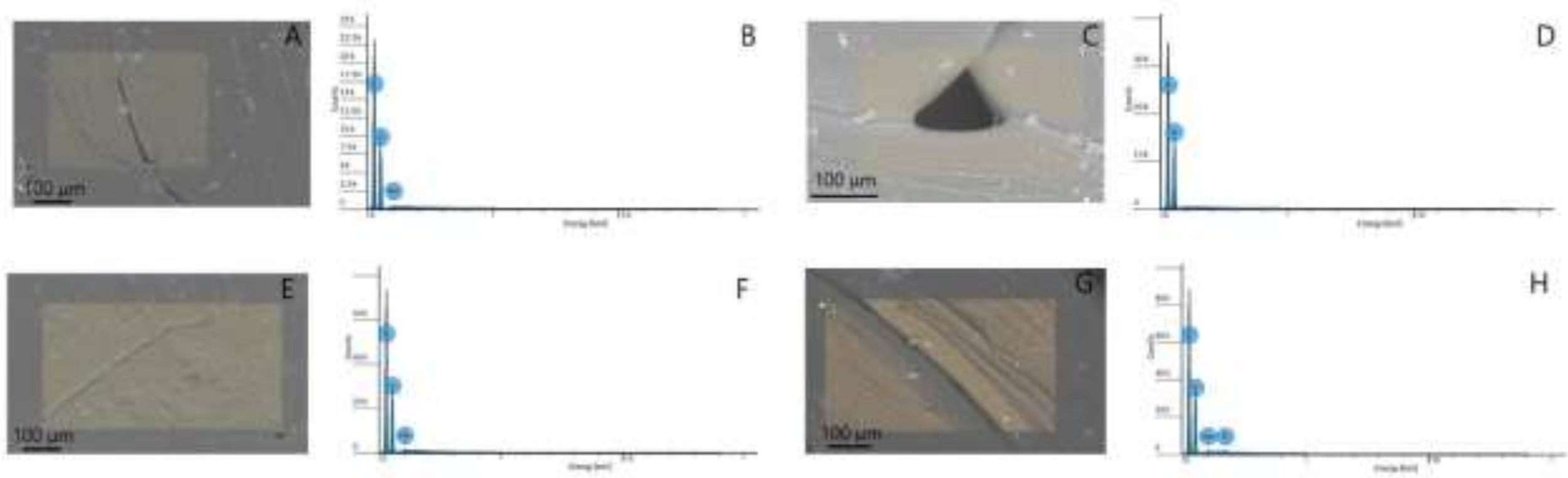

**Figure S2.** Representative SEM micrographs and corresponding EDX spectra of the 3D-printed carbon electrodes. (A, B) A1 Mini, (C, D) Creality, (E, F) X1-Carbon, and (G, H) HS. The highlighted regions in the SEM images indicate the areas selected for EDX analysis. The spectra confirm carbon and oxygen as the predominant elements in all samples, with only minor signals from trace elements.

*Tensile testing*

The tensile stress–strain curves obtained at stretching rates of 2.12e−3, 4.64e−3 and 1e-2 $s^{-1}$ for all investigated samples are reported in Figure S3.

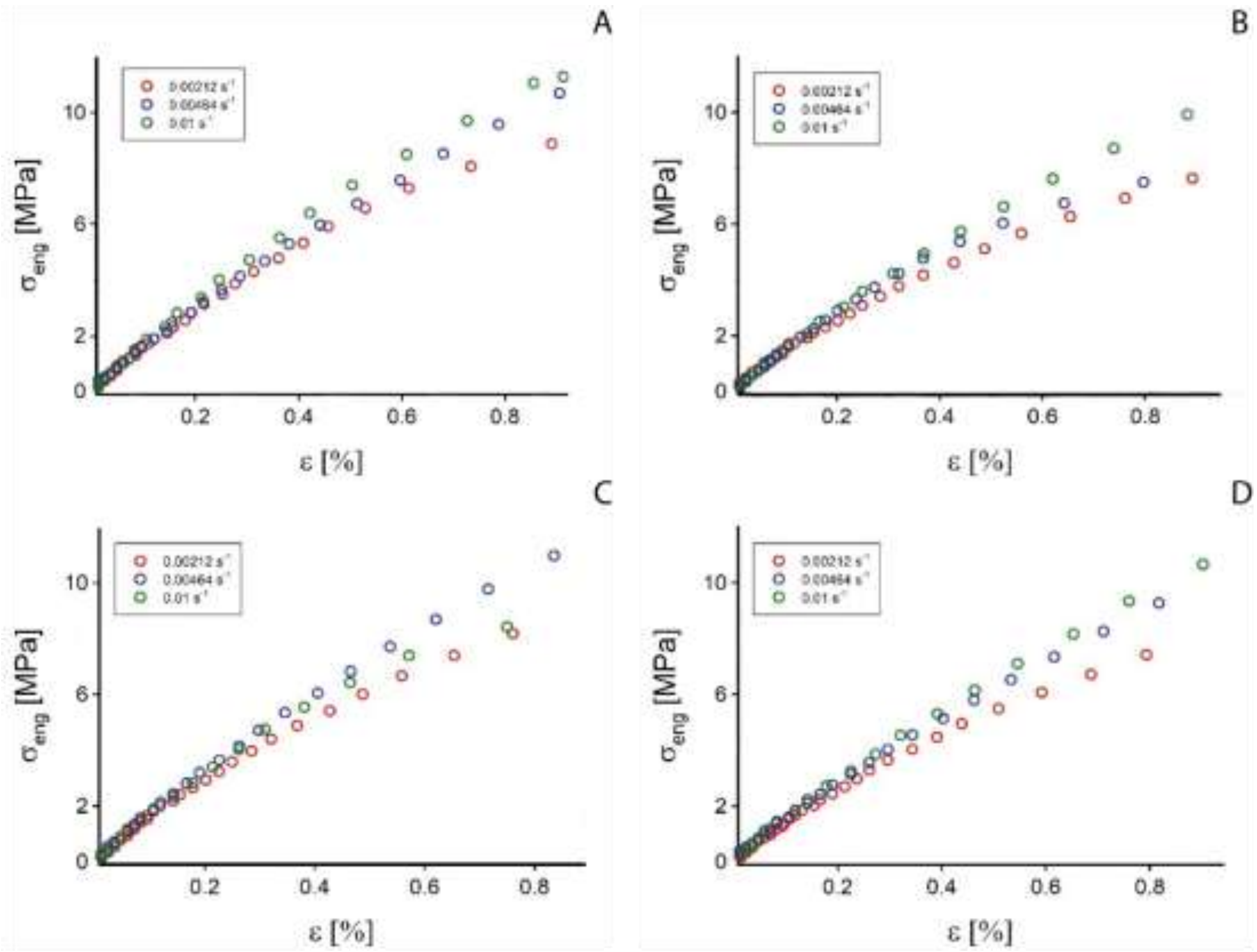


**Figure S3**. Stress-strain curve for X1-Carbon (A), A1 Mini (B), Creality (C), and HS (D).

*Electrochemical characterization of the 3D-printed electrodes using potassium ferricyanide*

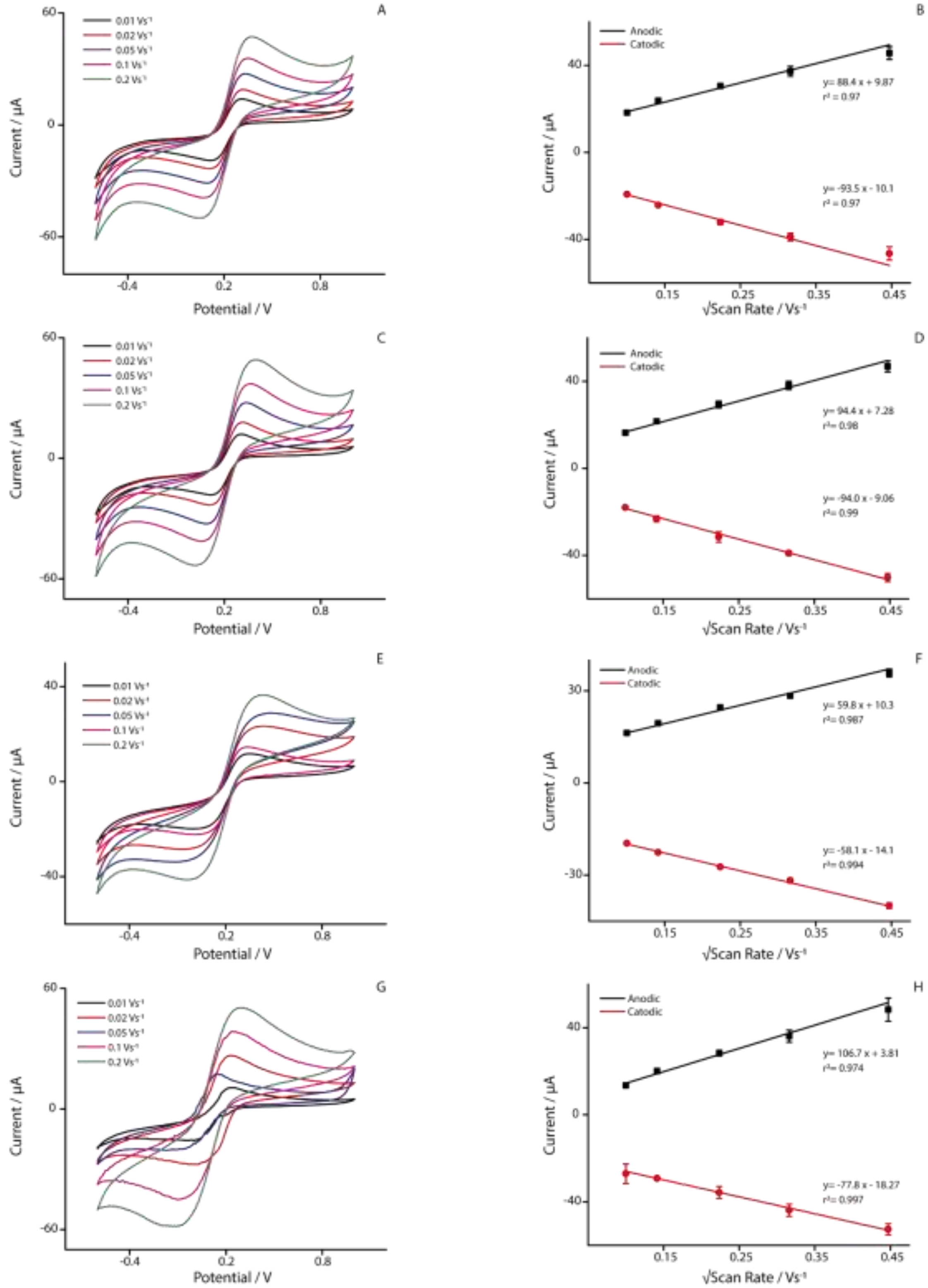


**Figure S4**. Cyclic voltammetry characterization of 3D-printed electrodes using 1 mM potassium hexacyanoferrate (III) as a redox probe. Cyclic voltammograms recorded at scan rates ranging from 0.01 to 0.2 V $s^{-1}$ and corresponding peak current vs. scan rate plots for the Bambu Lab X1-Carbon (A, B), A1 Mini (C, D), Creality (E, F), and HS (G, H). *Data are expressed as mean ± SD (n = 3).*

*Electrochemical characterization of the 3D-printed electrodes using hexammine ruthenium (III)*

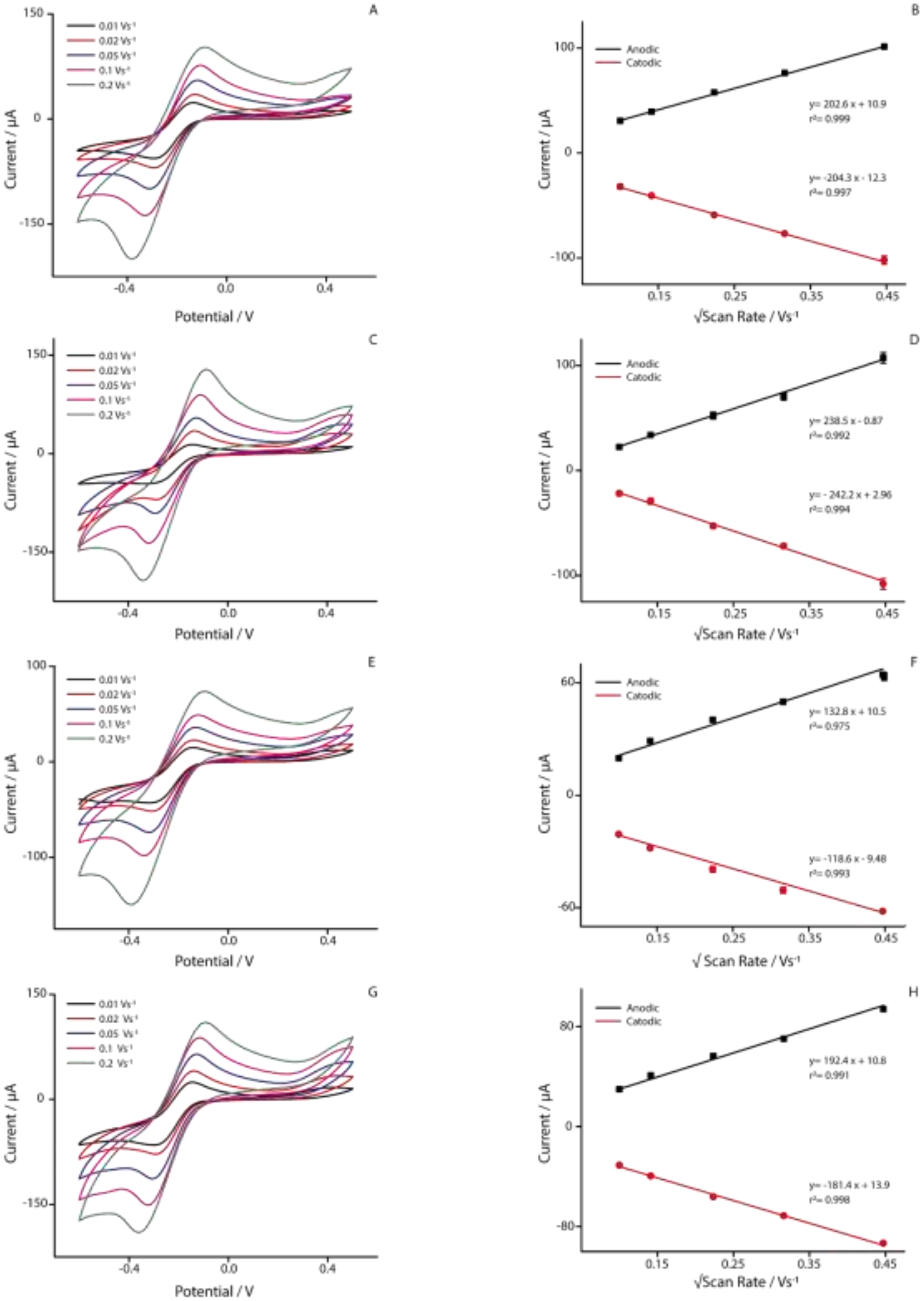


**Figure S5.** Cyclic voltammetry characterization of 3D-printed electrodes using 1 mM hexaammineruthenium(III) chloride as a redox probe. Cyclic voltammograms recorded at scan rates ranging from 0.01 to 0.2 V $s^{-1}$ and corresponding peak current vs. scan rate plots for the X1-Carbon (A, B), A1 Mini (C, D), Creality (E, F), and HS (G, H). Data are expressed as mean ± SD (n = 3).

### *Statistical Analysis*

Statistical analysis was performed using one-way analysis of variance (ANOVA), followed by Tukey's multiple comparisons test. All data were statistically analyzed using GraphPad Prism software version 8. $P < 0.05$ was considered significant.

**Table S2.** Relative standard deviation (RSD, %) of the electrochemical parameters obtained for the 3D-printed electrodes using potassium ferricyanide and hexaammineruthenium(III) as redox probes. The RSD values were calculated from three independent electrodes prepared using the respective printing platforms. ΔE, peak-to-peak separation; ECSA, electrochemically active surface area. The RSD of ECSA corresponds to that of the anodic peak current because ECSA was calculated directly from the anodic peak current using the Randles–Ševčík equation.

| **Potassium Ferricyanide** | **X1-Carbon** | **A1 Mini** | **Creality** | **HS** |
|---|---|---|---|---|
| ΔE (RSD) | 7.22% | 6.19% | 6.90% | 23.7% |
| Anodic peak current | 4.70% | 4.82% | 5.20% | 7.61% |
| Cathodic peak current | 2.76% | 6.44% | 2.29% | 10.3% |
| ECSA RSD | 4.70% | 4.82% | 5.20% | 7.61% |

| **Hexaamminoruthenium** | **X1-Carbon** | **A1 Mini** | **Creality** | **HS** |
|---|---|---|---|---|
| ΔE (RSD) | 6.25% | 4.03% | 3.69% | 1.88% |
| Anodic peak current | 1.64% | 1.76% | 15.9% | 1.13% |
| Cathodic peak current | 6.12% | 2.45% | 3.90% | 0.92% |
| ECSA RSD | 1.64% | 1.76% | 15.9% | 1.13% |

### *Quantitative analysis of surface material coverage from SEM images*

The surface material coverage of the 3D-printed electrodes was quantitatively evaluated from SEM micrographs using ImageJ software. SEM images acquired at the 1 mm scale were imported into ImageJ and converted to 8-bit grayscale images. The spatial scale was calibrated according to the scale bar provided in each SEM micrograph. A consistent region of interest (ROI) was then defined and saved as an ROI file to ensure that the same surface area was analyzed for all samples and that differences in the calculated coverage were not influenced by variations in the selected image area. Image thresholding was subsequently applied to distinguish the printed material from the darker void

regions between the deposited filaments. The thresholded images were analyzed using the ImageJ fraction measurement function to determine the area fraction corresponding to the dark void regions. For each electrode type, ten independent measurements were performed on electrodes fabricated using the corresponding 3D-printing platform. The percentage of void area was calculated directly from the thresholded images, while the surface material coverage was subsequently calculated as:

$$\text{Surface material coverage (\%)} = 100 - \text{Void area (\%)}$$

The resulting surface coverage percentages were used to quantitatively compare the degree of material deposition and surface continuity among the different printing platforms and were subsequently correlated with the corresponding electrochemical responses obtained using the two redox probes.

**Table S3.** Surface material coverage percentage of electrodes fabricated using different 3D-printing platforms. Values are expressed as mean ± standard deviation.

| Printer | Coverage percentage (%) |
|---|---|
| X1-Carbon | 94.11 ± 0.11 |
| A1 Mini | 90.00 ± 1.36 |
| Creality | 76.64 ± 4.64 |
| HS | 86.95 ± 0.81 |

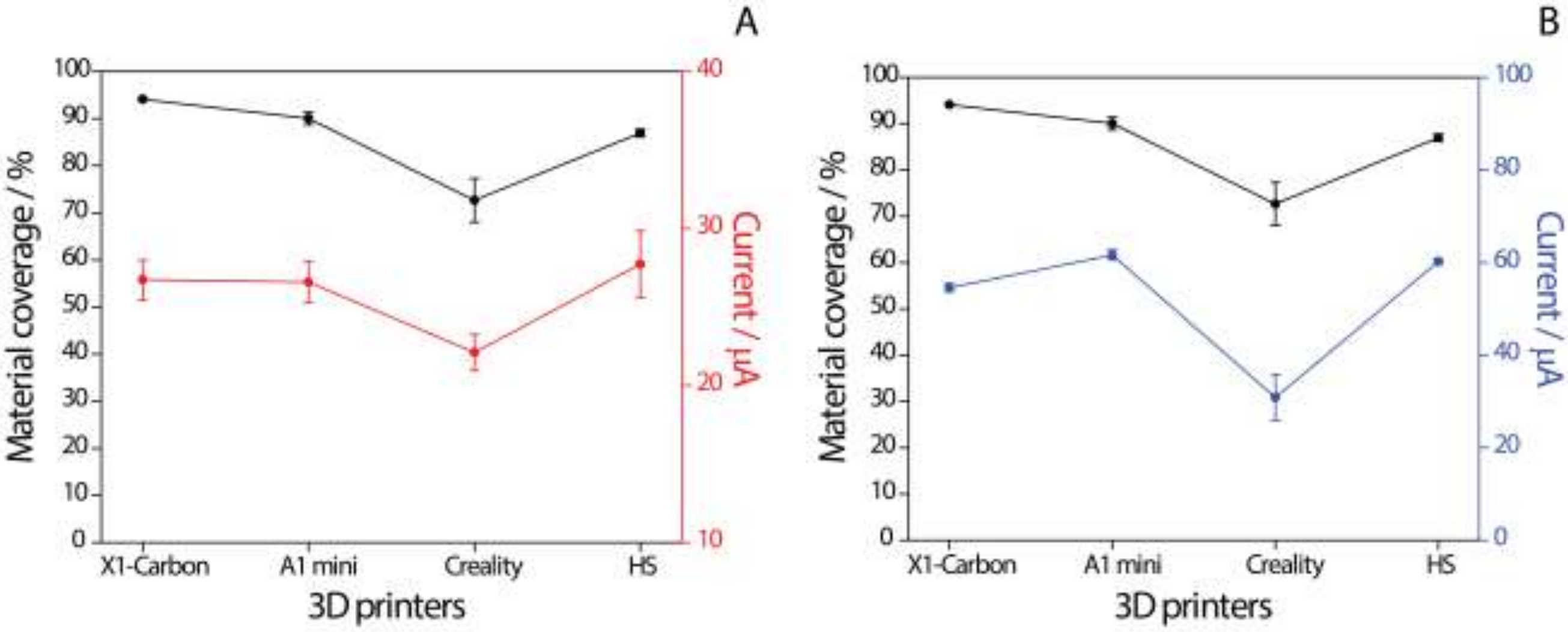


**Figure S6:** Relationship between the surface material coverage percentage and the anodic current response of the 3D-printed electrodes. Surface material coverage was quantified from SEM images by ImageJ analysis, with ten measurements performed for each electrode type. The anodic current response was obtained from cyclic voltammetric measurements and is expressed as mean ± SD (n = 3). (A) Potassium ferricyanide and (B) hexaammineruthenium(III) chloride. The plots illustrate the relationship between printing-dependent surface coverage and the corresponding electrochemical response of the different electrodes.

***Sustainability, practicality, and greenness of the 3D-printed electrodes***

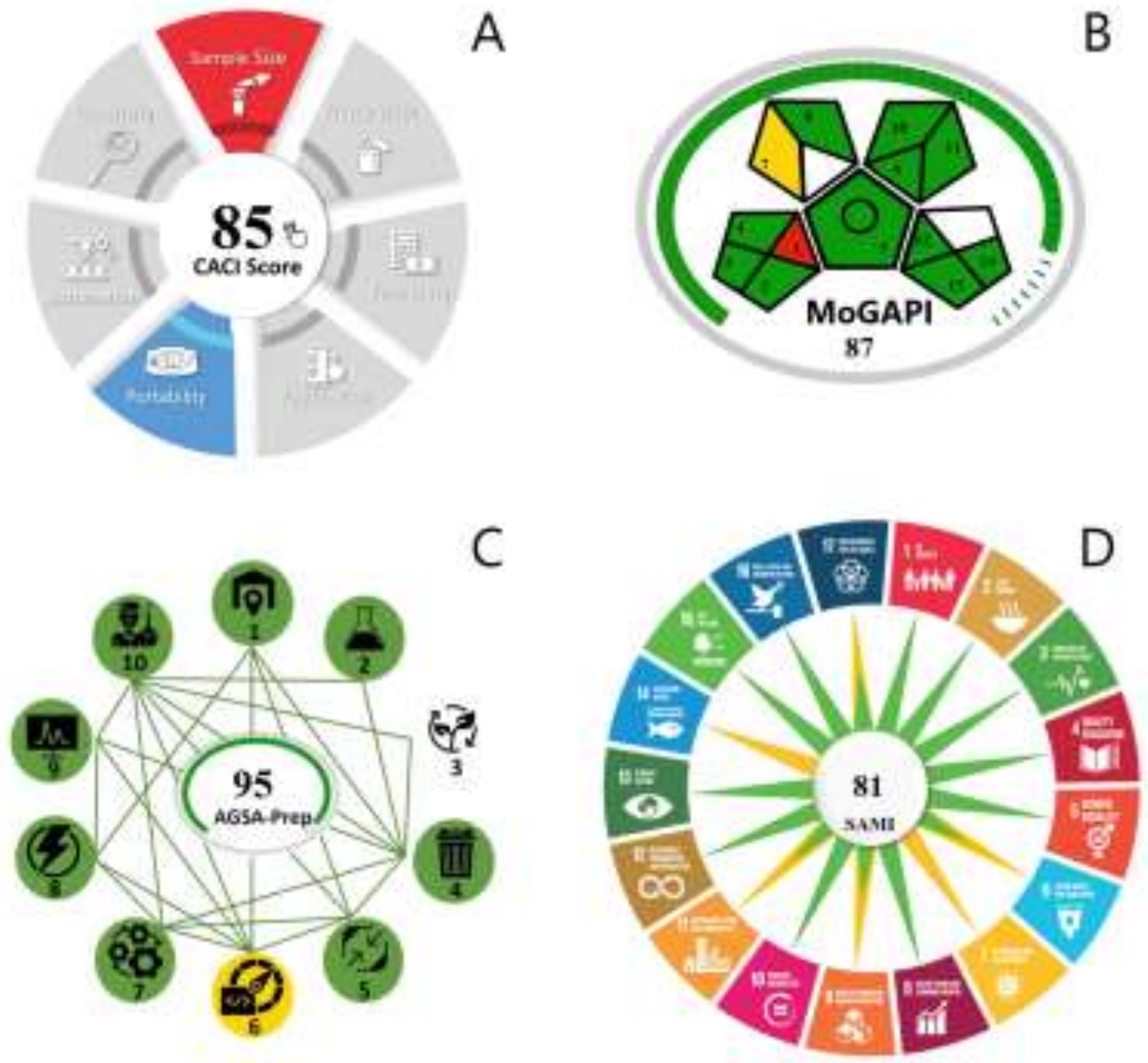


**Figure S7:** Overview of complementary metrics used to evaluate the sustainability, practicality, and greenness of the developed 3D-printed electrode platform. (A) Click Analytical Chemistry Index (CACI), assessing method simplicity and practicality across eight criteria (sensitivity, sample size, preparation, feasibility,

application, portability, automation), (B) Modified Green Analytical Procedure Index (MoGAPI), evaluating the greenness of each analytical step across 15 subsections, (C) AGSA-Prep (Analytical Green Star Area for Sample Preparation), assessing the greenness of the sample preparation step across 10 criteria including reusability, waste management, energy consumption, and operator protection, (D) Sustainability of Analytical Methods Index (SAMI), mapping the method's contribution across all 17 UN Sustainable Development Goals.
